# Short-term behavioural responses of Atlantic bluefin tuna to catch-and-release fishing

**DOI:** 10.1093/conphys/coac060

**Published:** 2022-09-02

**Authors:** Haley R Dolton, Andrew L Jackson, Alan Drumm, Lucy Harding, Niall Ó Maoiléidigh, Hugo Maxwell, Ross O’Neill, Jonathan D R Houghton, Nicholas L Payne

**Affiliations:** Department of Zoology, School of Natural Sciences, Trinity College Dublin, Dublin, D02 PN40, Ireland; Department of Zoology, School of Natural Sciences, Trinity College Dublin, Dublin, D02 PN40, Ireland; Marine Institute Newport, Fisheries Ecosystems Advisory Services, Furnace, County Mayo, F28PF65, Ireland; Department of Zoology, School of Natural Sciences, Trinity College Dublin, Dublin, D02 PN40, Ireland; Marine Institute Newport, Fisheries Ecosystems Advisory Services, Furnace, County Mayo, F28PF65, Ireland; Marine Institute Newport, Fisheries Ecosystems Advisory Services, Furnace, County Mayo, F28PF65, Ireland; Marine Institute Newport, Fisheries Ecosystems Advisory Services, Furnace, County Mayo, F28PF65, Ireland; School of Biological Sciences, Queen’s University Belfast, BT9 7DL, Northern Ireland; Department of Zoology, School of Natural Sciences, Trinity College Dublin, Dublin, D02 PN40, Ireland

## Abstract

Catch-and-release (C&R) angling is often touted as a sustainable form of ecotourism, yet the fine-scale behaviour and physiological responses of released fish is often unknown, especially for hard-to-study large pelagic species like Atlantic bluefin tuna (ABFT; *Thunnus thunnus*). Multi-channel sensors were deployed and recovered from 10 ABFTs in a simulated recreational C&R event off the west coast of Ireland. Data were recorded from 6 to 25 hours, with one ABFT (tuna X) potentially suffering mortality minutes after release. Almost all ABFTs (*n* = 9, including tuna X) immediately and rapidly (vertical speeds of ~2.0 m s^−1^) made powered descents and used 50–60% of the available water column within 20 seconds, before commencing near-horizontal swimming ~60 seconds post-release. Dominant tailbeat frequency was ~50% higher in the initial hours post-release and appeared to stabilize at 0.8–1.0 Hz some 5–10 hours post-release. Results also suggest different short-term behavioural responses to noteworthy variations in capture and handling procedures (injury and reduced air exposure events). Our results highlight both the immediate and longer-term effects of C&R on ABFTs and that small variations in C&R protocols can influence physiological and behavioural responses of species like the commercially valuable and historically over-exploited ABFT.

## Teaser

Catch-and-release fishing is seen as sustainable compared with overharvesting of fish, especially for commercially important species such as Atlantic bluefin tuna (ABFT). However, effects of this fishing on their behaviour and physiology are not fully understood. Here we show ABFTs are responding at two behavioural scales: immediately and hours post-release, with differing behavioural responses based on different capture and handling methods.

## Introduction

Catch-and-release (C&R) fishing is an increasingly popular practice, providing important social and economic benefits to communities while also being presented as sustainable (Policansky, 2002)**.** Atlantic Bluefin Tunas (ABFTs; *Thunnus thunnus*) are active, predatory fish historically over-exploited in commercial fishing and are a popular sport fish in C&R fisheries (Taylor *et al*., 2011) due to their size and power. The International Commission for the Conservation of Atlantic Tunas (ICCAT) manage ABFTs as two stocks (although there may be more genetically distinct spawning stocks in the NE Atlantic; Rooker *et al*., 2008, 2014; Rodríguez-Ezpeleta *et al*., 2019): as an ‘eastern stock’ and ‘western stock’, which is separated along the 45°W meridian (ICCAT, 2002; Block *et al*., 2005). The eastern stock is estimated to have declined to 33% of the historical levels in the early 2000s due to overfishing (Taylor *et al*., 2011) and in 2007 ICCAT began a stock rebuilding program, with the most recent assessment suggesting the eastern stock biomass is no longer decreasing (ICCAT, 2020). Despite the scientific assessment carried out by ICCAT, there remains a question surrounding the actual biomass of the ‘western’ or ‘eastern’ stock of ABFTs due to uncertainties surrounding the size of sexually mature individuals from each stock (Corriero *et al.*, 2020; Medina, 2020) and the reconciliation between the use of ‘eastern’ or ‘western’ areas by each stock for important life history events, with mixing between the stocks now widely accepted to occur (Block *et al.*, 2005; Stokesbury *et al.*, 2007; Rooker *et al.*, 2014; Hanke *et al.*, 2017; Horton *et al.*, 2020).

Recreational C&R is based on the assumption that released individuals recover from the interaction and contribute to future reproductive potential of the population (Cooke and Schramm, 2007; Donaldson *et al.*, 2008). If managed well, this approach can allow communities to benefit substantially, while helping to safeguard stocks (Cosgrove *et al.*, 2008). However, the sustainability of a C&R fishery for a particular species is underpinned ultimately by survivorship rates post-release including, but not limited to, suitability of fishing equipment, angler experience, species physiology and ecology and environmental conditions (Arlinghaus *et al.*, 2007; Cooke and Schramm, 2007; Davis, 2007; Brownscombe *et al.*, 2017). Consequently, methods to mitigate mortality during C&R events have been investigated in many teleost species (see Muoneke *et al.*, 2008; Brownscombe *et al.*, 2017, for a reveiw). However, due to the difficulty of studying large, marine species *in situ,* there remains significant uncertainties surrounding best C&R practices and mortality rates due to species-specific responses to capture (Bartholomew and Bohnsack, 2005; Muoneke *et al.*, 2008; Gallagher *et al.*, 2014). For example, survival rates of large sport fish appear highly variable, with reported survivorship rates varying from 22% to 100% for common thresher sharks (*Alopias vulpinus*; Sepulveda *et al.*, 2015), 90% for shortfin mako sharks (*Isurus oxyrinchus*; French *et al.*, 2015), 100% for yellowfin tunas (*Thunnus albacares*) and bigeye tunas (*Thunnus obesus*; Holland *et al.*, 1990) and 94–100% for ABFTs (Stokesbury *et al.*, 2011; Marcek and Graves, 2014). Such variation indicates areas for improvement and refinement regarding C&R practices with species-specific guidelines suggested to reduce mortality and sub-lethal effects (Cooke and Suski, 2005).

Survivorship rates represent the extreme endpoint metric of a C&R interaction. However, a range of sub-lethal impacts can have substantial consequences on the post-release fitness of individual fish and may have negative effects at the population level. These include physical injuries such as puncture wounds to the skin and sensitive tissues like the gills and eyes (reviewed in Brownscombe *et al.*, 2017) and possible impacts on suggested fitness measures such as elevated energy expenditure, increased predation risk and potential interruption of important activities such as feeding, growth and reproduction (Cooke *et al.*, 2002; Cooke and Suski, 2005; Raby *et al.*, 2013; Brownscombe *et al.*, 2014, 2017). Sub-lethal behavioural studies typically measure and describe stress proxies such as reflex impairment prior to fish release (Brownscombe *et al.*, 2013, 2017; McArley and Herbert, 2014), with the realized short-term physiological or behavioural fate of the animal post-release largely unknown. Recently, biologging devices have emerged as useful tools for measuring fine-scale behavioural, ecological and physiological parameters to assess biological function in fish (Cooke *et al.*, 2004; Watanabe *et al.*, 2008; Watanabe and Sato, 2008; Payne *et al.*, 2014) and to measure the post-release response in popular species targeted in C&R fisheries such as sharks (Whitney *et al.*, 2016, 2017; Brewster *et al.*, 2018; Hounslow *et al.*, 2019). Accelerometers, which can record high-resolution tri-axial acceleration, have been used to record body movements of bony fish after C&R to assess short-term impacts on behaviours such as swimming and predator avoidance (Brownscombe *et al.*, 2014, 2017; Holder *et al.*, 2020; LaRochelle *et al.*, 2021). Short-term effects on behaviour have been shown to indicate the long-term fate of fish, including survivability (Beitinger, 1990; Brownscombe *et al.*, 2014; Lennox *et al.*, 2018). However, despite their popularity as a sport fish, little is known about the short-term physiological and behavioural impact that C&R has on ABFTs beyond what can be inferred from lower resolution methods such as pop-off satellite archival tags (Stokesbury *et al.*, 2011; Marcek and Graves, 2014). We deployed accelerometers on 10 ABFTs captured during a simulated C&R event off the Northwest coast of Ireland. Our aim was to document the impact of C&R on the fine-scale behaviour of ABFTs immediately post-release and several hours thereafter.

## Methods

### Angling and tag attachment

Ten ABFTs were tagged under license from Health Products Regulatory Authority of Ireland between 2017 and 2020 (2017, *n* = 3; 2018, *n* = 4; 2019, *n* = 2 and 2020, *n* = 1) as part of a simulated recreational C&R event. As recreational fishing of ABFT is not permitted in Irish waters, a simulated C&R event was created whereby ABFTs were caught by anglers and brought to the boat for the purpose of a scientific procedure, as authorized within the Tuna Catch and Release Tagging (CHART) programme. A 120–200 lb line with 400 lb leader was used on a Class Rod (80–130 lb strength) to troll with squid lures on a spreader bar. Hooks (IO/O Mustad J hooks) were removed from fish immediately when brought on deck. All ABFTs were captured using this method within 20 km of the Donegal coastline, Ireland. Once close to the boat a lip hook was inserted into the mouth and out under the jaw to either secure the ABFT alongside the boat (*n =* 1) or to bring the ABFT on deck (*n* = 9). The use of lip hooks forms an important aspect of our simulated C&R study as they are used under the Irish CHART programme. Once on board ABFTs were placed on a padded mat with a deck hose inserted immediately in the mouth to ventilate the gills, a damp cloth placed over the eyes to reduce stress (following Block *et al.*, 2005) and the lip hook removed (lip hooking lasted ~10 seconds in duration). One fish was towed alongside the boat using a lip hook at 1–2 knots to ventilate the gills and was tagged while remaining partially submerged. Fork length (cm), half girth (cm), fish landing time, release condition, release time and position of capture were recorded. A floy tag was inserted near the second dorsal fin, and a tissue biopsy was taken from each fish as part of a wider study of ABFTs. A sterile fin clamp package containing a multi-channel data logger recording tri-axial acceleration at 20 Hz (resolution: 0.01 G) and 25 Hz (resolution: 2.0 G; Little Leonardo Corp. ORI1300 3MPD3GT, *n* = 7; and TechnoSmart AGM-1, *n* = 3, respectively) and depth at 1 Hz (resolution: 0.4 m and 0.1 m, respectively) was deployed on the second dorsal fin (Huveneers *et al*., 2018). Tri-axial acceleration was recorded at different frequencies due to logistical constraints. The package also contained a radio tag very high frequency transmitter (Advanced Telemetry Systems MM170B) and Satellite Position Only Tag (Wildlife Computers Model 258) to aid in recovery of the package (Supplementary Fig. S1; for package metrics, see Supplementary Table S1). As ABFTs are highly migratory, a galvanic timed release (rated for 1 or 2 days) was manually corroded down in a bucket containing seawater to 12–15 hours and used to hold the biologging package to the fin clamp. Manual corrosion of galvanic timed releases allowed the package to detach from the fin 6–25 hours later following submergence in seawater and to be successfully recovered. A second corrodible link in the fin clamp itself dissolved some 3–5 days later allowing all equipment to completely detach from the fish.

### Data analysis

Multi-channel data were initially analysed in Igor Pro 6.3 (WaveMetrics Inc., Portland, OR, USA) with the ‘Ethographer’ package (Sakamoto *et al.*, 2009). A low-pass filter was used to remove static (gravitational) from dynamic (body movement) components of the accelerometer data (following Watanabe and Takahashi, 2013). The clearest tailbeat signal on a dynamic wave was used in R (v. 4.0.3; R Core Team, 2020) to determine the dominant tailbeat frequency (TBF) for each individual (*n* = 9; 1 ABFT possibly suffered mortality and was excluded from this analysis). A loop function was created to run over every 12.5 minutes of data using an autoregression model to compute spectral density using the ‘stats’ package in base R. Trends in dominant TBF through time (as identified by the spectral density function) was visualized using the stat_smooth function in R to produce a generalized additive model (GAM) using the formula y ~ s(x) (family = loess).

Deployment locations were matched to 10 m resolution bathymetry data obtained from the Integrated Mapping for the Sustainable Development of Ireland’s Marine Resource in QGIS software (QGIS Development Team, 2018) to find release site depth. Tuna G was excluded from depth data analysis as the multi-channel data logger failed to record pressure. Depth data were analysed with a GAM using the formula y ~ s(x) (family = loess) to plot vertical speed data using ggplot in R. Depth data were also smoothed (*n* = 9) in Igor Pro 6.3 (WaveMetrics Inc., Portland, OR, USA) using an 8-point moving average smoother and the difference between two subsequent smoothed depth recordings were calculated producing vertical speeds whereby positive and negative values represent descents and ascents, respectively. Smoothed depth data from the initial 60 seconds post-release were plotted in R using ggplot as absolute terms and also as a proportion of the release site depth (%).

## Results

**Figure 1:**
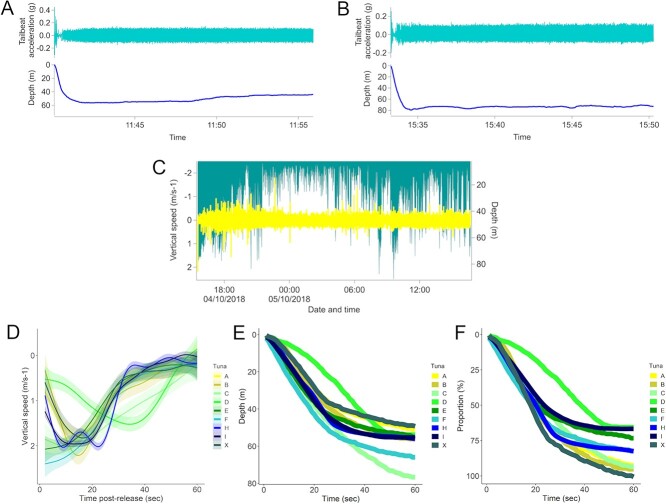
Representative examples of tailbeat signal and depth for two ABFTs (tuna A and C) during initial minutes post-release (**A**, **B**). Vertical descent speed (m s^−1^; yellow) and absolute depth (m; green) of tuna C during entire deployment (**C**). Vertical speed of ABFT during the initial minute post-release, represented by a GAM fitted to data (*n* = 9; **D**). Depth (m) of ABFT post-release (*n* = 9; **E**) and proportion of vertical space used by ABFT (%) during the first minute post-release (*n* = 9; **F**).

Between 2017 and 2020, large ABFTs ranging from 200–235 cm in length were tagged with multi-channel data loggers (*n* = 10) (Table 1) off the northwest coast of Ireland. Biologging packages stayed on the fin for 6–25 hours (Table 1; Supplementary Fig. S1), with packages detaching from the ABFT within 55 km (minimum straight-line distance) of the tagging site (Supplementary Fig. S2); fight time ranged from 13 to 24 minutes, and handling time (the duration from when ABFT were brought onto deck or secured alongside the boat to release) ranged from 2 to 9 minutes (Table 1). Initial tailbeat acceleration and depth data post-release revealed that the ABFTs undertook initial powered (associated with clear tailbeats) descents for several seconds, followed by a period of gliding that culminated with a tailbeat signal (displaying little variance between beats) within 1 minute post-release and horizontal swimming (Fig. 1A, B). The vertical speed of initial descent immediately post-release was noticeably higher (1.5–2.5 m s^−1^; Fig. 1C,D) than vertical descent speeds over the remaining time series, which were typically <0.5 m s^−1^ (Fig. S4). Following the initial rapid descent, the ABFTs began to reduce their vertical speed and approach near horizontal swimming (i.e. stop their descent) some 60 seconds post-release (Fig. 1D) at a depth of 45–80 m (Fig. 1E) using 66–98% of proportional water column (Fig. 1F). Tuna C, which remained partially submerged during the handling and tagging process, displayed a slightly slower return to near horizontal swimming during this time than the other ABFTs. Tuna D, which received a hooking injury to the eye, exhibited a noticeably different initial depth pattern to all other ABFTs, initially descending slowly and staying within 10 m of the surface and reaching its maximum vertical descent speed ~40 seconds after release (whereas others reached peak descent speeds <20 seconds after release; Fig. 1D).

**Table 1:** Tag attachment metadata for ten ABFT *T. thunnus* (deployment duration rounded to nearest hour)

Tuna ID	Date	Deploymentlatitude (°N)	Deploymentlongitude (°W)	Fork length(cm)	Fight time(mins)	Handlingtime (mins)	Deploymentduration (hrs)
A	18 October 2017	54.773	8.715	200	N/A	5	10
B	29 October 2017	54.901	8.693	235	N/A	4	7
C^a^	04 October 2018	54.544	8.850	205	20	9	25
D	04 October 2018	54.542	8.855	220	14	3	8
E	14 October 2018	54.513	8.855	220	17	2	16
F	31 October 2018	54.569	8.774	211	13	3	7
G	31 October 2019	54.490	8.719	222	19	4	11
H	31 October 2019	54.499	8.734	215	24	6	6
I	15 September 2020	54.576	8.828	207	19	5	16
X^b^	02 November 2017	54.892	8.649	200	N/A	7	N/A

^a^Not brought on deck, tagged in water.

^b^Possibly suffered mortality immediately after release.

Tuna X, which may have suffered mortality upon release (Fig. S3), had the longest time on deck (7 minutes; although was not a major outlier) and appeared to follow a similar pattern of descent as the other individuals, which lasted ~30 seconds, with a weak possible tailbeat signal lasting ~60 seconds. After this latter period, larger peaks in tailbeat amplitude were seen regularly through the time series for ~3 minutes with unusual accelerations, remaining relatively still on the seabed at a final depth of 65 m (Fig. S3). It is possible that the clamp had detached from the animal; however, the variable rate of descent was not consistent with such an event.

**Figure 2:**
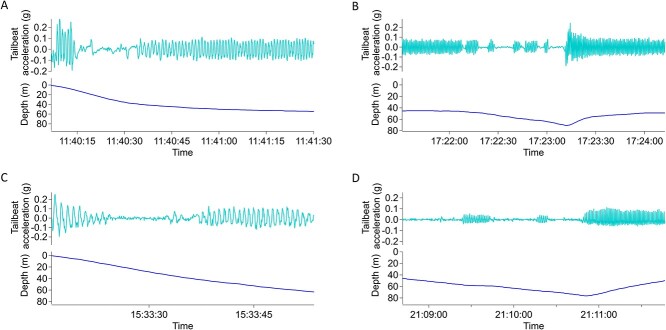
Examples of intermittent gliding behaviour shown in tailbeat acceleration (g) and depth (m) of tuna A and C immediately post-release (**A**, **B**, respectively) and some 5.5 hours post-release (**C**, **D**, respectively).

The percentage of proportional vertical space used within the water column by the ABFTs tagged on deck varied between 50–60% within approximately the first 20 seconds and 66–98% during the remaining 60 seconds post-release (Fig. 1F). Tuna D displayed a different descent response in relation to proportional vertical space use, using ~12.5% of the water column within the first 20 seconds (Fig. 1E,F). During the initial descent period, the ABFTs used intermittent tailbeat and gliding, a pattern also seen hours after release (Fig. 2; Fig. S5).

**Figure 3:**
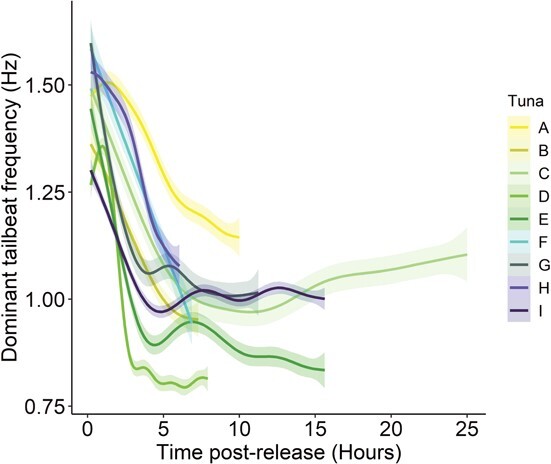
Dominant TBF over the entire deployment period for nine individual ABFT (**A**–**I**). To better visualize the data, dominant TBF was represented by a single data point every 12.5 minutes and smoothed using a GAM.

Spectral analysis over the entire deployment period of each ABFT revealed that dominant TBF was ~50% higher within the first few hours post-release (Fig. 3), and subsequently appeared to stabilize 0.8–1.0 Hz some 5–10 hours post-release. However, several traces (e.g. tunas A, F and H) continued to decrease for some of the shorter deployments (≤10 hours), suggesting dominant TBF had not stabilized at this time.

## Discussion

We used high-resolution accelerometery and depth data to demonstrate the behavioural responses of ABFTs to C&R angling. Individuals tagged on deck descended rapidly (i.e. 4-fold faster than subsequent descents) in the 20 seconds immediately after release, using 50–60% of the available water column, before gradually arresting their descent speeds and resuming horizontal swimming ~60 seconds post-release, at a depth of 50–80 m. Thereafter, the ABFTs swam with an elevated TBF for 3–7 hours before activity levels stabilized. These patterns were relatively consistent between individuals but punctuated by some notable exceptions. For example, one individual obtained an eye injury and another remained partially submerged during the handling process, with both exhibiting slightly different depth patterns than other ABFTs. Additionally, the individual with the longest time on deck may have suffered mortality.

The initial seconds post-release provided the first opportunity for the ABFTs to ventilate their gills after an anaerobic event and to metabolize waste products such as lactate, which may have accumulated during the fight time and on deck or aside vessel (Arends *et al.*, 1999; Cooke and Suski, 2005; French *et al.*, 2015). The rate of vertical ascent should be a strong determinant of total gas and heat exchange, given ABFTs are obligate ram ventilators, and so could be an important initial period of reoxygenation and heat exchange. Gleiss *et al.* (2019) found ABFTs use gliding descents upon release thought to agree with Weihs’ two-stage locomotion model allowing conservation of energy on descents during gravity-assisted locomotion (Weihs, 1973). However, during the first 60 seconds post-release the ABFTs used between 66% and 98% of the available water column, and while ABFTs are negatively buoyant, the descent was powered by intermittent locomotion despite a marked increased locomotor effort thought to not be needed to overcome the hydrostatic force of a gas bladder during depth changes of ABFTs (Gleiss *et al.*, 2019). The extent to which this initial powered descent represents an ‘escape’ or ‘stress’ response (e.g. triggered by adrenaline production) versus a recovery from anaerobic exercise could be an important question for future work and to identify lower limits of water depth over which ABFTs can be safely released.

As with the relatively similar responses of ABFTs in the first minute after release, there was a strikingly consistent pattern of TBF being ~50% higher post-release than TBFs reached 5–10 hours later. While TBF for some of the shorter deployment durations seemed to still be declining when the biologging tags detached from the animal, the first 5–10 hours post-release is characterized by higher activity levels and increased mechanical output, with potential baseline activity levels reached thereafter. While we do not have data extending beyond 25 hours, a previous study from the east Atlantic (Gleiss *et al.*, 2019) showed similar trends of TBF to our study with tailbeat frequencies remaining elevated for several hours post-release (approximately ≥1.5 Hz) and gradually declining to <1 Hz some 6 hours post-release establishing a potential TBF baseline that was similar several days later. The physiological driver of this extended period of elevated activity is unknown, but has also been reported in sharks following a C&R event (Whitney *et al.*, 2016), and will represent a large increase in energy expenditure of animals subjected to C&R compared with those that do not (since metabolic rate scales exponentially with swimming speed (Weihs, 1977; Jacoby *et al.*, 2015; Ryan *et al.*, 2015).

A variety of C&R factors are known to influence fish fitness and mortality post-release (see Brownscombe *et al.*, 2017, for a review). For example, common thresher sharks caught in recreational fisheries display significantly elevated lactate levels with increased fight time (Sepulveda *et al.*, 2015), with air exposure also known to increase lactate and mortality levels (Lennox *et al.*, 2015; Brownscombe *et al.*, 2017; Mohan *et al.*, 2020). Our study represented a simulated C&R event with experienced anglers that bring ABFTs to the boat quickly and efficiently. However, most ABFTs were assisted onto deck by using lip hooks, a practice that is often advised against for C&R fisheries, and briefly exposed to air during the tagging process prior to the hose being introduced to the mouth for irrigation (Danylchuk *et al.*, 2008; Brownscombe *et al.*, 2017). The one ABFT that was tagged in the water exhibited a slightly different depth response to other ABFTs, so it could be instructive to further explore the extent to which bringing ABFTs on deck influences subsequent behaviour and physiology. Further refinements in biologging techniques and addition of sensors such as video cameras could also enhance detection of important post-release behaviours such as active resumption of feeding, which is indicative of good physiological status (Del Caño et al., 2021) after capture.

Developing species-specific guidelines can aid in the development of a sustainable C&R fishery for a species that is vulnerable to overexploitation. Our study demonstrated a notable similarity in initial depth use in the first minute post-release and a decline in dominant TBF several hours later. However, an individual hooked in the eye and an individual who remained partially submerged during tagging displayed different responses to other ABFTs in this study. These two exceptions could serve to motivate further exploration of how variations in C&R procedures influence subsequent welfare outcomes for ABFTs. With the number of C&R programmes of ABFTs in the north-east Atlantic likely to increase, careful monitoring of fishing techniques and the species-specific behavioural responses to C&R will help safeguard sustainable fishing of the commercially vulnerable and valuable ABFT.

## Author contributions

 Overall conception by N.L.P., N.O.M., A.D. and H.R.D. Fieldwork undertaken by H.R.D., N.L.P., A.D., R.O.N., L.H., J.D.R.H. and H.M. Data analysis undertaken by H.R.D. and N.L.P. Writing led by H.R.D. and N.L.P. with contribution from all authors. All authors read, edited and discussed the manuscript.

## Funding

Research conducted by H.R.D in this publication was funded by the Irish Research Council under award number (GOIPG/2019/4197). A.L.J. was funded by the Irish Research Council grant (IRCLA/2017/186). L.H. and N.L.P. were supported by Science Foundation Ireland (18/SIRG/5549) and Marine Institute (SERV-18-FEAS-079, SERV-19-FEAS-071 & SERV-20-FEAS-074b). A.D., N.O.M., H.M. and R.O.N. were funded by the European Maritime and Fisheries Fund—Sustainable Fisheries Scheme A3: Innovation.

(19.SF.A.03). Fieldwork was conducted under license from the Health Products Regulatory Authority of Ireland (#AE19136/P127).

## Supplementary Material

suppl_data_coac060
